# *Dendrobium officinale* polysaccharide ameliorates polycystic ovary syndrome by modulating the MAPK/ERK signaling pathway

**DOI:** 10.3389/fphar.2026.1786264

**Published:** 2026-08-21

**Authors:** Xueyu Qin, Yongyan Lu, Peng Zeng, Guanyou Huang, Heng Luo, Shuyun Zhao

**Affiliations:** 1 Guizhou Medical University Clinical Medical College, Guiyang, China; 2 Gynecology Department, Anshun People’s Hospital, Anshun, China; 3 Guizhou Natural Products Research Center, Guiyang, China

**Keywords:** *Dendrobium officinale*, MAPK/ERK signaling pathway, ovarian function, polycystic ovary syndrome, traditional Chinese medicine

## Abstract

**Introduction:**

Polycystic ovary syndrome (PCOS) is one of the most common endocrine and metabolic disorders affecting women of reproductive age. *Dendrobium officinale* polysaccharides (DOP) have shown multiple pharmacological activities, but their therapeutic effects and underlying mechanisms in PCOS remain incompletely understood.

**Methods:**

A dehydroepiandrosterone (DHEA)-induced mouse model of PCOS was established to evaluate the effects of DOP. Estrous cyclicity, ovarian histopathology, serum hormone levels, RT-qPCR, and Western blot analyses were performed to assess reproductive function, endocrine changes, and MAPK/ERK-related signaling.

**Results:**

DOP treatment restored estrous cyclicity, improved ovarian morphology, and partially normalized serum luteinizing hormone, follicle-stimulating hormone, and testosterone levels. In addition, DOP reduced ERK1/2 phosphorylation and downregulated the expression of CYP17A1 and CYP19A1.

**Conclusion:**

DOP ameliorated reproductive and endocrine abnormalities in a DHEA-induced mouse model of PCOS. The findings suggest that modulation of MAPK/ERK signaling may contribute to the biological effects of DOP, although further mechanistic studies are required to establish causal relationships.

## 1 Introduction

Polycystic ovary syndrome (PCOS), also known as Stein–Leventhal syndrome, is one of the most prevalent endocrine and metabolic disorders affecting adolescent girls and women of reproductive age. It is a heterogeneous condition characterized by reproductive, endocrine, and metabolic dysfunction. The major clinical features include menstrual irregularities, hyperandrogenism, obesity, hirsutism, chronic anovulation-associated infertility, and polycystic ovarian morphology. In addition to its reproductive manifestations, PCOS is frequently associated with insulin resistance, dyslipidemia, and an increased risk of long-term cardiometabolic complications, imposing a substantial burden on women’s health and quality of life (Lizneva et al., 2016). The pathophysiology of PCOS is complex and multifactorial, involving interactions among neuroendocrine, metabolic, inflammatory, and ovarian factors. Dysregulation of the hypothalamic–pituitary–ovarian (HPO) axis is considered a central feature of the disorder and is characterized by increased gonadotropin-releasing hormone (GnRH) pulse frequency and an elevated luteinizing hormone/follicle-stimulating hormone (LH/FSH) ratio, which contribute to ovarian androgen excess and ovulatory dysfunction (Pratama et al., 2024). Current management strategies include lifestyle modification and pharmacological interventions, such as combined oral contraceptives, insulin-sensitizing agents, and anti-androgen therapies. Surgical approaches are reserved for selected cases and are used less frequently. However, existing treatments primarily target symptom control rather than the underlying pathophysiological mechanisms and may be associated with adverse effects or limited long-term efficacy. Consequently, the identification of safe and effective therapeutic alternatives remains an important area of research (Tang et al., 2023).

Polycystic ovary syndrome (PCOS) is one of the most prevalent endocrine–metabolic disorders affecting adolescents and women of reproductive age worldwide. It is a heterogeneous condition characterized by reproductive, endocrine, and metabolic dysfunction. Common clinical manifestations include menstrual irregularities, hyperandrogenism, obesity, hirsutism, anovulatory infertility, and polycystic ovarian morphology. The pathophysiology of PCOS is multifactorial and involves complex interactions among neuroendocrine, metabolic, inflammatory, and ovarian factors. Dysregulation of the hypothalamic–pituitary–ovarian (HPO) axis is considered a central feature of the disorder and is characterized by increased gonadotropin-releasing hormone (GnRH) pulse frequency and an elevated luteinizing hormone/follicle-stimulating hormone (LH/FSH) ratio, which contribute to ovarian androgen excess, follicular arrest, and ovulatory dysfunction.

Current management strategies for PCOS primarily include combined oral contraceptives, insulin-sensitizing agents, and anti-androgen therapies, with surgical interventions reserved for selected cases. However, these treatments largely focus on symptom management rather than addressing the underlying pathophysiological mechanisms of the disorder. Moreover, their long-term use may be limited by adverse effects, variable patient responsiveness, and suboptimal clinical outcomes. Consequently, there remains a pressing need to develop safer, more effective, and mechanism-based therapeutic approaches for PCOS.

Globally, PCOS affects approximately 6%–10% of women of reproductive age, with reported prevalence estimates in China ranging from 5% to 21% depending on the diagnostic criteria and study population (Liu J. et al., 2021; Zhang J. et al., 2024). Beyond its high prevalence, PCOS is associated with an increased risk of infertility, miscarriage, and a range of long-term metabolic and cardiovascular complications, imposing a substantial burden on women’s health. To date, no curative therapy is available. Current treatment strategies primarily rely on pharmacological interventions and, in selected cases, surgical procedures. However, these approaches largely focus on symptom management and may be limited by adverse effects, variable therapeutic responses, and suboptimal long-term outcomes (Domecq et al., 2013). Therefore, the development of safer, more effective, and mechanism-based therapeutic strategies remains an important unmet clinical need in PCOS management. Traditional Chinese medicine (TCM) has attracted increasing attention as a complementary therapeutic approach for PCOS because of its multitarget characteristics and generally favorable tolerability profile. Accumulating experimental and clinical evidence suggests that various herbal formulations and natural products may improve ovarian morphology and function, normalize endocrine disturbances, alleviate insulin resistance, and reduce chronic inflammation associated with PCOS (Wang et al., 2020; Hager et al., 2019; Liu M. et al., 2021; Venkatesan et al., 2023; Zhao et al., 2021). Mechanistic studies have implicated several signaling pathways in these effects, including PI3K/Akt, MAPK/ERK, and TLR4/NF-κB signaling, although the precise molecular mechanisms remain incompletely understood (Gao et al., 2021; Diamanti-Kandarakis and Dunaif, 2012; Otoo et al., 2023).

Among these pathways, dysregulation of PI3K/Akt signaling has been closely linked to insulin resistance, metabolic dysfunction, and impaired ovarian physiology in PCOS (Cui et al., 2022; Gong et al., 2020; Lin et al., 2024; Zhang et al., 2022). Likewise, activation of inflammatory pathways, particularly TLR4/NF-κB signaling, contributes to the chronic low-grade inflammatory state that characterizes the disorder (Otoo et al., 2023; de Oliveira et al., 2017; Brenjian et al., 2020). Collectively, these findings suggest that modulation of key endocrine, metabolic, and inflammatory signaling networks may represent a promising strategy for PCOS management.

*Dendrobium officinale* Kimura & Migo, the dried stem of an orchid species widely used in traditional Chinese medicine, has long been employed as a medicinal and functional food with Yin-nourishing properties (Huang et al., 2023). Pharmacological studies have demonstrated a broad spectrum of biological activities, including antioxidant, immunomodulatory, antidiabetic, and antitumor effects (Rong et al., 2024). Among its bioactive constituents, *Dendrobium officinale* polysaccharides (DOP) have attracted particular attention because of their regulatory effects on glucose and lipid metabolism, oxidative stress, and insulin sensitivity (Zhang Y. et al., 2024; Liu et al., 2020; Okoro et al., 2023; Wang et al., 2018). Experimental evidence suggests that these effects may be associated with the modulation of multiple signaling pathways, including IRS2/PI3K/Akt, cAMP–PKA, and Akt/FoxO1 signaling (Zhang Y. et al., 2024; Liu et al., 2020; Okoro et al., 2023; Wang et al., 2018). In addition, structural and functional studies have indicated potential interactions between DOP and immune-related targets such as TLR4, supporting its immunomodulatory properties (Yang et al., 2024). Emerging clinical and preclinical evidence further suggests that *Dendrobium officinale*–based interventions may exert beneficial effects on reproductive function and metabolic homeostasis, highlighting their potential relevance in the management of PCOS (Feng et al., 2022).

Previous studies have demonstrated that MAPK/ERK signaling plays an important role in ovarian function, steroidogenesis, and endocrine regulation, and its dysregulation has been implicated in the pathogenesis of PCOS (Feng et al., 2022; Zhao et al., 2025; Teede et al., 2023; Lan et al., 2015). Based on this evidence, we hypothesized that *D. officinale* polysaccharides (DOP) may ameliorate PCOS-associated reproductive and endocrine abnormalities through modulation of ERK-related signaling. Therefore, this study was designed to evaluate the therapeutic effects of DOP in a dehydroepiandrosterone (DHEA)-induced mouse model of PCOS and to determine whether DOP treatment is associated with changes in ERK phosphorylation and the expression of steroidogenesis-related genes.

## 2 Materials and methods

### 2.1 Experimental animals

Ninety 3-week-old female mice were purchased from Henan Kerbes Biotechnology Co., Ltd. (License No. SCXK [Henan] 2020–0005). All animals were provided with sterilized standard diet and autoclaved drinking water, while housed under controlled conditions (22 °C ± 2 °C; relative humidity 45%–55%; 12-h light/dark cycle) at the Guizhou Provincial Natural Products Research Center. After a 7-day acclimation period, experiments were initiated. The study protocol was approved by the Ethics Committee of Guizhou Medical University (Approval No. 2303462).

### 2.2 Reagents and instruments

The following instruments were employed to conduct the experiments: ultracentrifuge (USA, Beckman L7-65), cryogenic benchtop centrifuge (Germany, Eppendorf 5810R), protein electrophoresis system (USA, Mini-PROTEAN® Electrophoresis Cell), gel imaging system (USA, Bio-radGelDoc XR), Nucleic Acid Protein Analyzer (USA, Thermo Nanodrop 2000), real-time PCR instrument (Switzerland, Lightcy-cler 480II), PCR instrument (Germany, eppendorf MC NEXUS GSX1), inverted fluorescence microscope (Olympus, IX71), automatic microplate reader (USA, BIOTEK Elx808), laboratory ultrapure water machine (Shanghai, China, Best-S15 FV), rotary evaporator (Shanghai Hujia Instrument Equipment Co., Ltd., RE-52C), electric blast drying oven (Tianjin, China, WGL-230B), and biological safety cabinet (ESCO. EBA-5UDG-0). The ELISA kit was purchased from Nanjing Institute of Bioengineering. Anti-ERK1/2, Anti-p-ERK1/2, Anti-ATF2, Anti-MEK1, Anti-p-MEK1, and Anti-p38 antibodies were purchased from Wuhan Saiwell Biotechnology Co., Ltd.

### 2.3 Extraction of DOP-associated components from dendrobium officinalis

**FIGURE 1:**
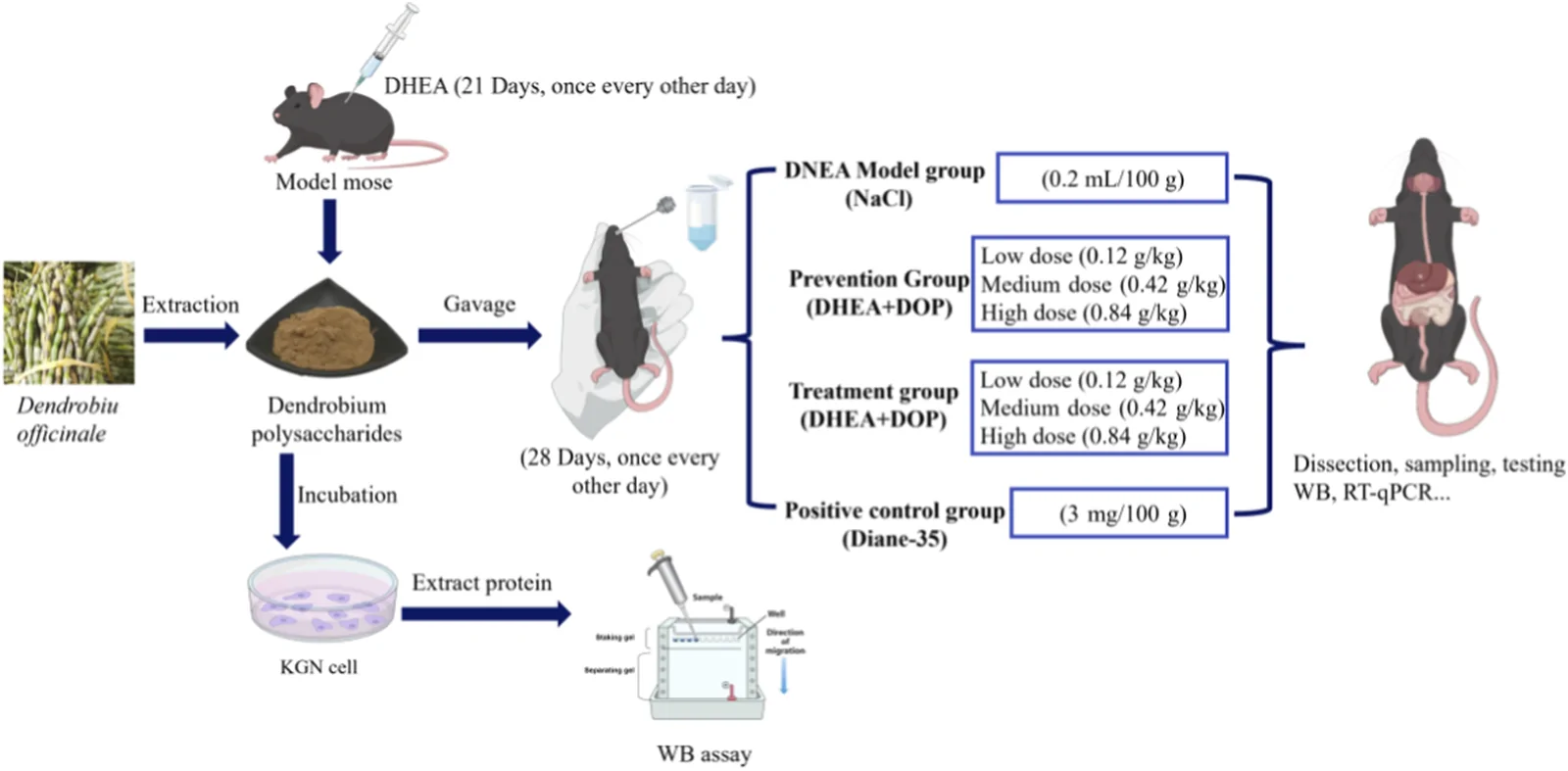
Full-text experimental flowchart.

The herbal material was supplied by Guizhou Anlong Materia Medica Biotechnology Co., Ltd. The botanical source was the dried stem (pharmacopoeial drug name: Dendrobii Officinalis Caulis; Chinese name: Tiepishihu) of *D. officinale* Kimura & Migo (Orchidaceae). Pharmacognostic/morphological identification was performed by the Guizhou Provincial Natural Products Research Center, and a voucher specimen was deposited for future reference (Voucher specimen No. LT00241200861). Fresh D. officinale stems (1 kg) were dried at 60 °C, pulverized, and passed through a 60-mesh sieve to obtain 120 g of powder. The powder was defatted with 5 volumes (w/w) of anhydrous ethanol, stirred, and kept at 25 °C for 48 h, followed by vacuum filtration through 4 μm filter paper to collect the residue. The residue was placed in a filter bag and extracted with water at a 20-fold (w/w) ratio in a 70 °C water bath for 3 h, repeated three times, and then subjected to ultrasonic extraction at 70 °C for 1 h, repeated three times. The combined extracts were vacuum-filtered (4 μm) and concentrated to a syrupy consistency using rotary evaporation at 60 °C. Subsequently, three volumes (v/v) of absolute ethanol were added under stirring, and the mixture was kept at 25 °C for 24 h to precipitate polysaccharides. The precipitate was collected by vacuum filtration (4 μm), dissolved in a minimal volume of water, and lyophilized to yield 20.4 g of DOP. The extraction yield was calculated as polysaccharide mass/powder mass, giving 20.4/120 = 17%. The flowchart outlining this procedure is shown in Figure 1.

### 2.4 Animal model construction

Prepubertal female C57BL/6J mice were randomized into model and control groups. To induce the PCOS model, the mice received daily dorsal subcutaneous injection with dehydroepiandrosterone (DHEA) dissolved in 0.2 mL of sterile sesame oil at a dosage of 6 mg/100 g body weight for 28 consecutive days. Control group mice were administered an equivalent volume of sterile sesame oil at the same site. During the treatment, body weight changes were regularly monitored. Upon completion of the treatment, the estrous cycle was monitored through vaginal smear, the ovarian tissue morphology was examined via HE staining to identify polycystic alterations, and serum sex hormone levels were quantified to validate successful model construction. DOP doses were chosen by integrating the dosage information for *Dendrobium officinale* in the Pharmacopoeia of the People’s Republic of China, body-surface-area–based dose-conversion principles recommended in animal-experiment guidelines, and commonly used effective dose ranges of DOP reported in the literature. Low/middle/high dose levels were designed to balance translational relevance, safety, and assessment of dose–response.

### 2.5 DOP administration

The mice were further divided into 3 groups: prevention group, DOP treatment group, and positive control group. In prevention group, the mice received daily gavage of 0.1 mL distilled water containing different doses of DOP during the 28-day DHEA injection period for PCOS model construction. This group was subdivided into 0-dose prevention group (0DPG): 0 g/kg/day DOP, low-dose prevention group (LDPG): 0.21 g/kg/day DOP, medium-dose prevention group (MDPG): 0.42 g/kg/day DOP, and high-dose prevention group (HDPG) 0.84 g/kg/day DOP.

In DOP treatment group, following the 28-day DHEA injection period for PCOS model construction, the mice were administered DOP at specified doses via daily gavage for 28 days. This group was subdivided into 0 treatment group (0DTG): 0 g/kg/day DOP, low-dose treatment group (LDTG): 0.21 g/kg/day DOP, medium-dose treatment group (MDTG): 0.42 g/kg/day DOP, and high-dose treatment group (HDTG): 0.84 g/kg/day.

In the positive control group, mice were administered Diane-35 dissolved in 0.1 mL distilled water at a dose of 3 mg/100 g body weight for 28 days, following the 28-day DHEA injection period for PCOS model construction. Upon completion of the experiment, anesthetized mice received an intraperitoneal injection of uratan, and samples were collected according to the requirements of subsequent assays.

### 2.6 Hematoxylin-eosin (HE) staining

After sample collection, the unilateral ovary and a section of the liver from mice in each group were fixed in 4% paraformaldehyde (PFA) for 4–6 h, followed by immersion in 20% sucrose solution overnight at 4 °C. The ovarian tissues were embedded in paraffin and serially sectioned at a thickness of 8 μm. The fixed liver tissues were embedded in OCT medium (Tissue-Tek), rapidly frozen in liquid nitrogen, stored at −80 °C overnight, and then sectioned into 7 μm slices using a Leica cryostat for subsequent HE staining.

### 2.7 Vaginal smear

To monitor the estrous cycle, mice from each group underwent daily vaginal smears at 10:00 AM for two consecutive weeks. This procedure was performed following the completion of PCOS modeling induced by DHEA and subsequent treatments with Diane-35 or various doses (low, medium, and high) of DOP.

The normal murine estrous cycle comprises 4 consecutive stages: preestrus, estrus, postestrus, and estrus. Staging was determined based on the presence and proportion of nucleated cells, keratinized epithelial cells, and leukocytes observed in the smears.

### 2.8 Enzyme-linked immunosorbent assay

According to the instructions of the ELISA kit, serum LH, FSH, and testosterone (T) levels, and LH/FSH ratio were measured for mice in all experimental groups.

### 2.9 RT-qPCR

Ovarian tissues from each group were rapidly frozen in liquid nitrogen and pulverized. Total RNA was extracted using the TRIzol RNA Extraction Kit according to the manufacturer’s protocol, and then reverse transcribed into cDNA. According to the prescribed reaction system, qPCR reaction was carried out. The relative mRNA expression levels of ERK1/2 (MAPK1), CYP17A1, and CYP19A1 were quantified by RT-qPCR.

### 2.10 Western blot

Total protein was extracted from ovarian tissues and ovarian granulosa cells, and protein concentration was quantified using BCA assay. The proteins were separated by SDS-PAGE and then transferred to a membrane (200 V, 220 mA, 2 h). After blocking with 5% BSA, the membrane was incubated with primary antibody on a shaker at 4 °C overnight. Following 3 washes with 1 × TBST, the membrane was incubated with secondary antibody for 2 h at a room temperature. Protein bands were visualized and scanned to determine the protein expression levels of ERK1/2, phosphorylated ERK1/2 (p-ERK1/2), MEK1, phosphorylated MEK1 (p-MEK1), ATF2, and p38 by Western blot analysis.

### 2.11 Statistical analysis

Statistical analysis was performed using IBM SPSS software. Multiple t-tests and one-way tests of variance were used for intergroup comparison. Data are presented as mean ± standard deviations. Each experiment was independently repeated 3 times (n = 3). A p-value <0.05 was considered statistically significant.

## 3 Results

### 3.1 DOP effectively restores the estrous cycle in mice

**FIGURE 2:**
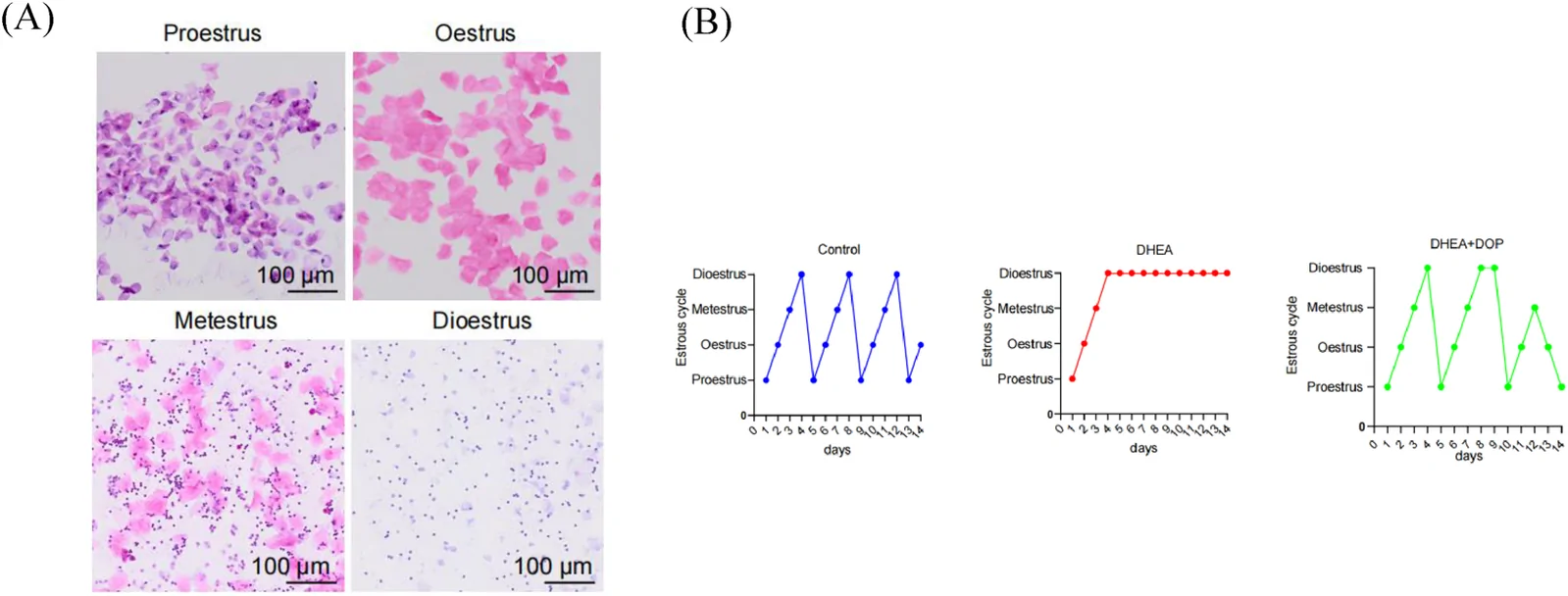
**(A)** HE staining of vaginal secretory cell smears from mice; **(B)** Estrous cycle monitoring in the control group, the DHEA model group and the DHEA + DOP group. Image enlarged 200 ×. Scale bar = 100 μm.

Estrous cycle disruption is a hallmark manifestation of PCOS. The typical estrous cycle comprises four consecutive stages: proestrus, oestrus, metestrus and diestrus. The detection methods include morphological examination of reproductive organs, behavioral observation, and vaginal cytology. In this experiment, HE-stained vaginal smears were used to assess the estrous cycle across experimental groups. Figure 2A illustrates a normal estrous cycle progression of mice. As shown in Figure 2B, compared with the control group, the PCOS model group exhibited a significantly disordered estrous cycle. Specifically, 90% of the mice remained in the diestrus phase for prolonged periods, while the remaining 10% showed acyclic transitions among other stages without regular periodicity. Notably, intervention with DA and varying doses (low, medium, and high) of DOP after model establishment resulted in a 92.3% normalization rate of the estrous cycle.

### 3.2 Analysis of mouse body weight and ovarian histopathology

**FIGURE 3:**
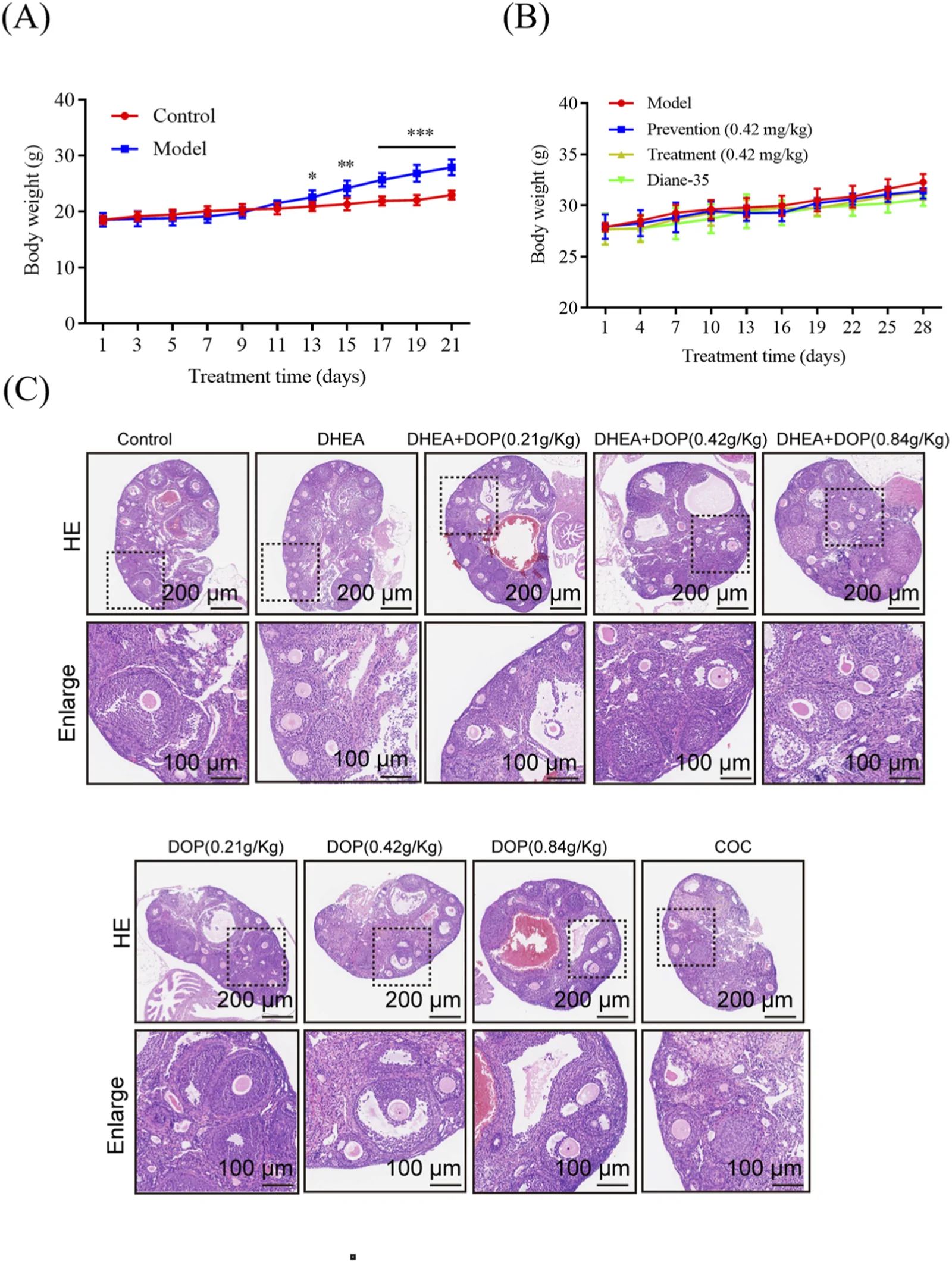
**(A)** Body weight changes of mice in each group during modeling. **(B)** Body weight changes of mice in each group during drug administration. **(C)** HE stained sections of mouse ovarian tissues in each experimental group. Image enlarged 400 ×. Scale bar = 50 μm. Compared with the model group, ^*^ *P <* 0.05, ^**^ *P <* 0.01, *P <* 0.001.

As shown in Figure 3A, statistical analysis of body weight during modeling revealed that mice in the model group exhibited significantly higher body weights compared with the control group, with statistically significant from Day 13 (p < 0.05). During the subsequent 28-day treatment period as shown in Figure 3B, mice in the prevention, treatment, and positive control (Dietin-35) groups all exhibited varying degrees of weight reduction relative to the model group. These results suggest that the treatment effectively mitigated body weight gain.

Further, to evaluate the effect of DOP on ovarian histopathology in mice from each group, HE staining was carried out. As shown in Figure 3C, the control group, exhibited relatively normal ovarian tissue architecture, with orderly follicular development, follicles at various stages, a limited number of corpus lutea, and thick, regularly arranged granulosa cell layers. In contrast, mice in the DHEA-induced PCOS model group displayed significant cystic dilatation, a reduced number of granulosa cell layers, markedly decreased follicles and corpus lutea, and noticeable proliferation of theca interna and stromal cells. In both the prevention group (DHEA + DOP) and the treatment group (DOP), the number of cystic follicles decreased and the follicular morphology gradually recovered toward normal. These results indicates that DOP can effectively ameliorate ovarian damage in PCOS mice.

### 3.3 ELISA analysis of LH, FSH, and T levels in mouse serum

**FIGURE 4:**
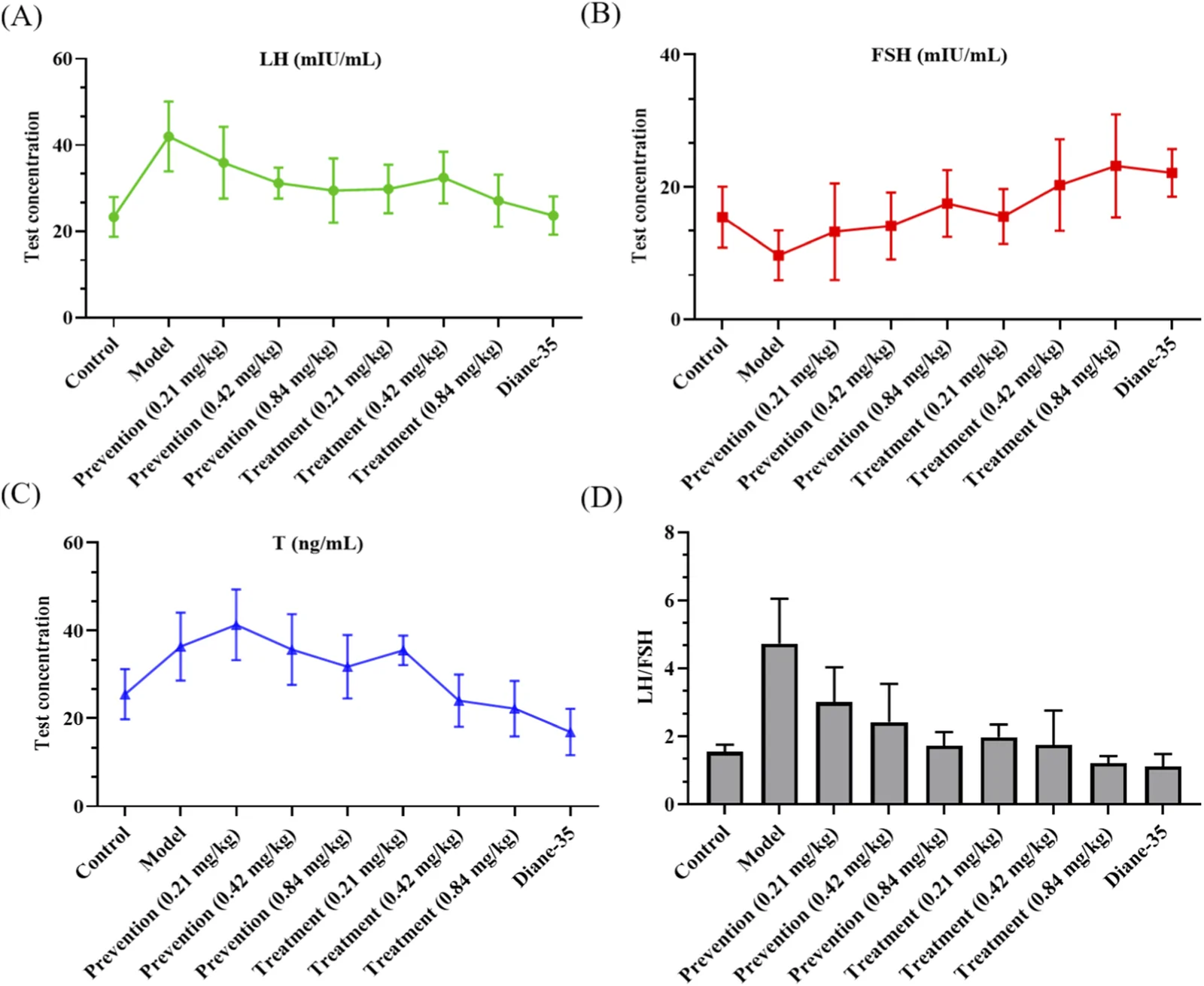
Serum levels of LH, FSH, T, LH/FSH in mice in each experimental group. **(A)** LH concentration; **(B)** FSH concentration; **(C)** T concentration; **(D)** LH/FSH ratio.

Serum levels of LH, FSH and T are critical biomarkers for assessing ovarian function. The LH, FSH and T values of each group changed significantly. As illustrated in Figures 4A,C, mice in the model group exhibited significantly elevated LH and T levels. Following intervention with various doses of DOP, both LH and T levels showed marked improvement. As shown in Figure 4B, compared with the blank group, FSH level notably decreased in the model group. However, after DOP administration in prevention and treatment groups, FSH values increased to varying degrees. The LH/FSH ratio, a key indicator for PCOS progression, exceeded 3 IU/L, indicative of an imbalance between LH and FSH. As shown in Figure 4D, the LH/FSH ratio in the model group was the highest, significantly above 3. In contrast, this ratio decreased after DOP prevention and treatment, with the blank group showing the lowest value. These findings suggest that DOP prevention and treatment can effectively modulate the LH/FSH imbalance, thereby ameliorating of PCOS development. The raw data are provided in Supplementary Material 1.

### 3.4 RT–qPCR analysis of ERK1/2, CYP17A1, and CYP19A1 expression

**FIGURE 5:**
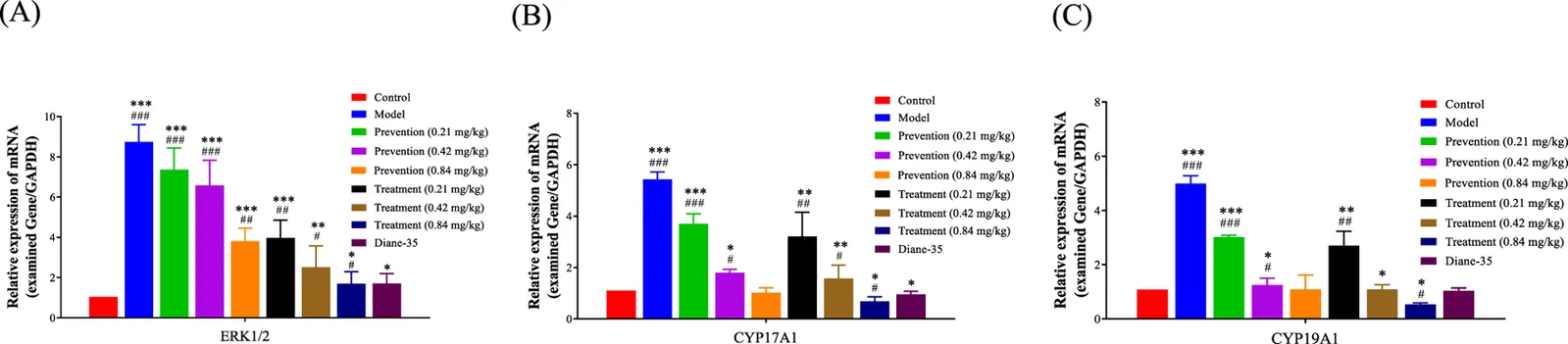
mRNA expression in mouse ovarian tissues determined by RT-qPCR in each group. **(A)** ERK1/2; **(B)** CYP17A1; **(C)** CYP19A1. Compared with the blank group, ^*^ *P <* 0.05, ^**^ *P <* 0.01, *P <* 0.001. Compared with the Diane-35 group, ^#^ *P <* 0.05, ^##P^ *<*0.01, ^###^ *P <* 0.001.

To investigate whether DOP treatment was associated with alterations in MAPK/ERK signaling, RT–qPCR was performed to evaluate the mRNA expression levels of ERK1/2, CYP17A1, and CYP19A1 in ovarian tissues from each experimental group. As shown in Figures 5A–C, the expression levels of all three genes were significantly increased in the model group compared with the control group (P < 0.05). Following preventive or therapeutic administration of DOP, the expression levels of ERK1/2, CYP17A1, and CYP19A1 were reduced to varying degrees relative to the model group (P < 0.05). These findings indicate that DOP treatment was associated with reduced expression of ERK1/2 and steroidogenesis-related genes.

### 3.5 Western blot analysis of MAPK/ERK signaling proteins

**FIGURE 6:**
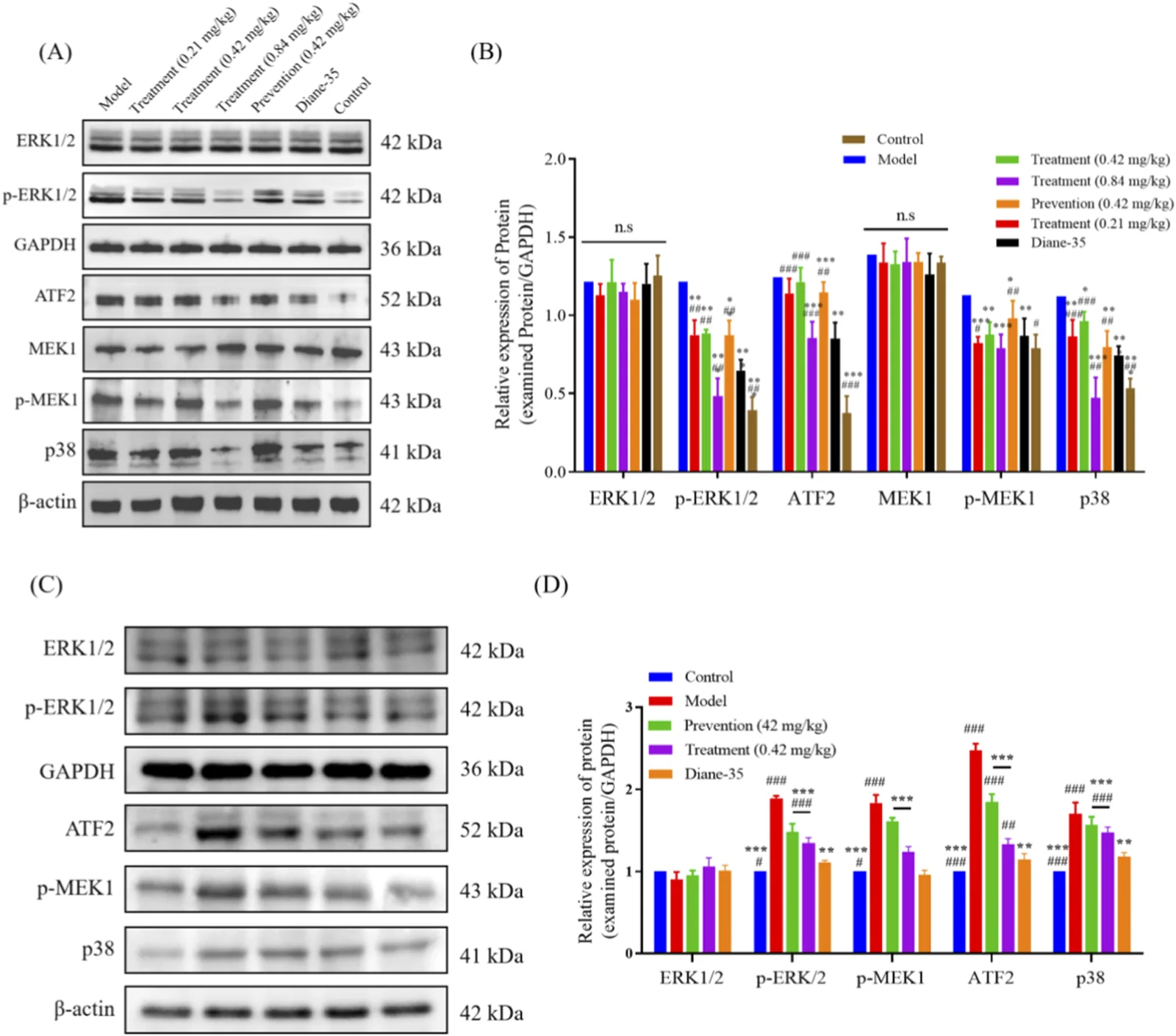
Western blot analysis of protein expression in mouse ovarian tissue and ovarian granulosa cells. **(A,B)** Immunoblots and quantitative analysis of proteins associated with MAPK signaling in mouse ovarian tissues. **(C,D)** Immunoblots and quantitative analysis of proteins associated with MAPK signaling. These findings suggest that modulation of MAPK/ERK signaling may contribute to the therapeutic effects of DOP. in KGN of mouse granulosa cells. Compared with the model group, ^*^ *P <* 0.05, ^**^ *P <* 0.01, *P <* 0.001. Compared with the Diane-35 group, ^#^ *P* < 0.05, ^##^P < *0.01*, ^###^ *P <* 0.001.

Western blot analysis was performed to determine the protein expression levels of ERK1/2, phosphorylated ERK1/2 (p-ERK1/2), MEK1, phosphorylated MEK1 (p-MEK1), ATF2, and p38 in mouse ovarian tissues and KGN granulosa cells. As shown in Figures 6A,B (ovarian tissues) and Figures 6C,D (KGN cells), the protein levels of p-ERK1/2, ATF2, p-MEK1, and p38 were the highest in DHEA-induced model group and the lowest in control (blank) group (p < 0.01). However, following preventive or therapeutic intervention with DOP, the expression levels of these proteins were reduced to varying degrees. Notably, the high-dose DOP group showed stronger inhibition than the Diane-35 group (p < 0.01). In contrast, the total protein levels of ERK1/2 and MEK1 showed no significant differences among the experimental groups. These findings demonstrate that DOP treatment was associated with reduced phosphorylation of ERK-related signaling proteins, whereas the total expression levels of ERK1/2 and MEK1 remained unchanged.

## 4 Discussion

In this study, we evaluated the effects of *Dendrobium officinale* polysaccharides (DOP) in a dehydroepiandrosterone (DHEA)-induced mouse model of PCOS. DOP treatment improved estrous cyclicity, alleviated ovarian histopathological abnormalities, partially normalized serum hormone levels, and was associated with alterations in MAPK/ERK-related signaling. Together, these findings suggest that DOP may exert beneficial effects on reproductive and endocrine dysfunction in PCOS. Further studies are required to validate the molecular targets involved, establish causal mechanisms, and evaluate the translational potential of DOP in clinical settings.

Disruption of the estrous cycle is a characteristic reproductive feature of PCOS. In the present study, DOP treatment was associated with improvements in estrous cyclicity in DHEA-induced PCOS mice. Given the concurrent normalization of serum hormone profiles, these effects may be related, at least in part, to improved endocrine homeostasis (Qi et al., 2020). Notably, mice receiving prophylactic DOP administration exhibited relatively stable estrous cycles throughout the study period, whereas those in the therapeutic group showed a gradual recovery of cyclicity beginning around Day 12. These findings suggest that DOP may exert both preventive and therapeutic effects on reproductive dysfunction associated with PCOS.

Consistent with the observed improvements in reproductive function, histological examination demonstrated a reduction in cystic follicles and a partial restoration of ovarian morphology following DOP treatment. These findings indicate that DOP may alleviate ovarian structural abnormalities associated with PCOS, although the cellular mechanisms underlying these effects remain to be clarified (Shen et al., 2025). In addition, DOP treatment significantly improved serum LH, FSH, and testosterone levels, suggesting a beneficial effect on endocrine disturbances in the DHEA-induced PCOS model. Given the central role of hormonal dysregulation in PCOS, the normalization of these endocrine parameters may contribute to the observed improvements in ovarian morphology and estrous cyclicity (Yuan et al., 2025). However, further studies examining hypothalamic–pituitary–ovarian axis regulation are required to determine whether DOP directly influences neuroendocrine function.

RT–qPCR and Western blot analyses demonstrated that DOP treatment was associated with reduced ERK1/2 mRNA expression and decreased ERK1/2 phosphorylation, whereas the total protein levels of ERK1/2 remained relatively unchanged. These findings suggest that DOP may influence MAPK/ERK signaling, at least in part, through modulation of ERK phosphorylation rather than by altering total ERK1/2 protein expression.

ERK signaling is a major downstream component of the MAPK pathway and plays important roles in regulating cellular proliferation, differentiation, inflammatory responses, and metabolic homeostasis. Previous studies have suggested that dysregulated ERK signaling contributes to ovarian dysfunction and endocrine abnormalities associated with PCOS. Previous studies have suggested that aberrant ERK activation may contribute to insulin-signaling dysfunction through the serine phosphorylation of proximal signaling mediators, including insulin receptor substrate-1 (IRS-1) (Ye et al., 2022). In addition, altered ERK signaling has been reported in experimental models of PCOS and may be associated with endocrine and metabolic abnormalities characteristic of the disorder (Ye et al., 2022; Zhang et al., 2016).

In the present study, DOP treatment was associated with reduced ERK1/2 expression and phosphorylation together with improvements in ovarian morphology, estrous cyclicity, and hormone profiles. These findings suggest that modulation of MAPK/ERK signaling may contribute to the biological effects of DOP in the DHEA-induced PCOS model. However, causal relationships between ERK signaling and the observed reproductive and endocrine improvements remain to be established through additional mechanistic studies.

Given that DOP is a polysaccharide extract, its molecular-weight distribution, structural heterogeneity, batch-to-batch consistency, and pharmacokinetic characteristics may vary across species. Therefore, the human-equivalent dose estimated from the present animal study should be regarded only as a preliminary reference rather than a clinical recommendation. Future translational studies should establish standardized manufacturing procedures and quality-control criteria, including molecular-weight profiling and structural fingerprinting, together with comprehensive safety, toxicology, and PK/PD evaluations. As a logical next step toward clinical translation, a standardized DOP preparation could be investigated in an early-phase clinical study to assess safety, tolerability, and preliminary pharmacodynamic effects, thereby providing a basis for subsequent efficacy trials in patients with PCOS (Francis et al., 2025).

Several limitations of this study should be acknowledged. First, although DOP treatment was associated with modulation of ERK signaling, the present study primarily established an association rather than a causal relationship. Pharmacological rescue experiments and genetic manipulation studies were not performed to determine whether modulation of ERK signaling is required for the therapeutic effects of DOP. Second, ovarian histology was evaluated mainly qualitatively, and comprehensive histomorphometric analyses were not conducted. Third, functional validation of steroidogenesis, including enzyme activity assays and targeted steroid profiling, as well as systematic assessments of metabolic outcomes and safety, were beyond the scope of the present study. Furthermore, as a polysaccharide extract, DOP was not subjected to detailed structural characterization, molecular-weight profiling, or batch-to-batch consistency evaluation. These limitations should be addressed in future studies to further elucidate the molecular mechanisms of DOP and facilitate its translational development for PCOS.

Future studies should prioritize establishing causal relationships between MAPK/ERK signaling and the biological effects of DOP, for example, through pharmacological rescue experiments or genetic approaches designed to restore ERK activity. Such studies would help determine whether modulation of ERK signaling is required for the observed therapeutic effects in the present PCOS model. In parallel, further efforts should focus on the comprehensive characterization of DOP, including its molecular-weight distribution, structural features, and batch-to-batch consistency, as well as the identification of biologically active fractions. These investigations will be important for improving reproducibility, facilitating mechanistic studies, and supporting the future translational development of DOP.

In summary, DOP exerted beneficial effects in a DHEA-induced mouse model of PCOS, including restoration of estrous cyclicity, improvement of ovarian histopathology, and normalization of serum hormone profiles. To place these findings within a broader pathophysiological framework, MAPK/ERK signaling has been implicated in both endocrine and metabolic abnormalities associated with PCOS. Previous studies have suggested that dysregulated ERK activity may contribute to insulin-signaling dysfunction through the phosphorylation of insulin receptor substrate-1 (IRS-1), thereby linking metabolic and reproductive disturbances characteristic of the disorder. In addition, ERK signaling has been associated with cellular proliferation, differentiation, and tissue remodeling processes that are relevant to ovarian function.

In the present study, DOP treatment was associated with reduced ERK1/2 expression and phosphorylation, together with improvements in ovarian morphology and endocrine parameters. These observations are consistent with the hypothesis that modulation of MAPK/ERK signaling may contribute to the biological effects of DOP. However, because insulin resistance, IRS-1 signaling, and downstream metabolic outcomes were not directly assessed, the specific relationship between ERK modulation and metabolic improvement remains speculative and warrants further investigation. More broadly, PCOS is increasingly recognized as a multifactorial disorder involving complex interactions among endocrine, metabolic, inflammatory, and ovarian signaling networks. The therapeutic effects of DOP may therefore involve coordinated regulation of multiple pathways beyond MAPK/ERK signaling alone, which will require further mechanistic studies to elucidate.

Overall, the present findings suggest that DOP may ameliorate reproductive and endocrine abnormalities in experimental PCOS, with modulation of MAPK/ERK signaling representing a potential mechanism that warrants further mechanistic investigation.

## Data Availability

All data are included in the article and supplementary materials. Additional data are available from the corresponding author upon reasonable request.
