# Supplementary material for: Dendrobium officinale polysaccharide ameliorates polycystic ovary syndrome by modulating the MAPK/ERK signaling pathway
Source: Front Pharmacol. 2026 Aug 21;17:1786264. doi: 10.3389/fphar.2026.1786264 (PMC13541706; doi:10.3389/fphar.2026.1786264)
Supplement: Supplementary file 1 [file Table1.docx]

S Table1

T levels T (ng/mL)

| Grouped | Mice 1 | Mice 2 | Mice 3 | Mice 4 | Mice 5 |
| --- | --- | --- | --- | --- | --- |
| Control | 20.645 | 24.14 | 31.83 | 22.8083 | 28.2682 |
| Model | 41.765 | 34.47 | 43.285 | 23.945 | 38.55 |
| Prevention (0.21 mg/kg) | 47.04 | 35.605 | 37.935 | 45.612 | 40.4205 |
| Prevention (0.42 mg/kg) | 41.43 | 30.005 | 33.21 | 38.45 | 35.4925 |
| Prevention (0.84 mg/kg) | 39.735 | 30.015 | 25.585 | 29.6 | 33.9565 |
| Treatment (0.21 mg/kg) | 33.37 | 37.505 | 39.2 | 32.145 | 35.455 |
| Treatment (0.42 mg/kg) | 25.76 | 18.615 | 23.075 | 33.435 | 19.47 |
| Treatment (0.84 mg/kg) | 30.065 | 19.2 | 13.55 | 25.995 | 22.51 |
| Diane-35 | 12.185 | 21.525 | 22.995 | 11.275 | 16.57 |

S Table2

FSH (mIU/mL)

| Grouped | Mice 1 | Mice 2 | Mice 3 | Mice 4 | Mice 5 |
| --- | --- | --- | --- | --- | --- |
| Control | 20.645 | 24.14 | 31.83 | 22.8083 | 28.2682 |
| Model | 41.765 | 34.47 | 43.285 | 23.945 | 38.55 |
| Prevention (0.21 mg/kg) | 47.04 | 35.605 | 37.935 | 45.612 | 40.4205 |
| Prevention (0.42 mg/kg) | 41.43 | 30.005 | 33.21 | 38.45 | 35.4925 |
| Prevention (0.84 mg/kg) | 39.735 | 30.015 | 25.585 | 29.6 | 33.9565 |
| Treatment (0.21 mg/kg) | 33.37 | 37.505 | 39.2 | 32.145 | 35.455 |
| Treatment (0.42 mg/kg) | 25.76 | 18.615 | 23.075 | 33.435 | 19.47 |
| Treatment (0.84 mg/kg) | 30.065 | 19.2 | 13.55 | 25.995 | 22.51 |
| Diane-35 | 12.185 | 21.525 | 22.995 | 11.275 | 16.57 |

S Table3

LH (mIU/mL)

| Grouped | Mice 1 | Mice 2 | Mice 3 | Mice 4 | Mice 5 |
| --- | --- | --- | --- | --- | --- |
| Control | 20.645 | 24.14 | 31.83 | 22.8083 | 28.2682 |
| Model | 41.765 | 34.47 | 43.285 | 23.945 | 38.55 |
| Prevention (0.21 mg/kg) | 47.04 | 35.605 | 37.935 | 45.612 | 40.4205 |
| Prevention (0.42 mg/kg) | 41.43 | 30.005 | 33.21 | 38.45 | 35.4925 |
| Prevention (0.84 mg/kg) | 39.735 | 30.015 | 25.585 | 29.6 | 33.9565 |
| Treatment (0.21 mg/kg) | 33.37 | 37.505 | 39.2 | 32.145 | 35.455 |
| Treatment (0.42 mg/kg) | 25.76 | 18.615 | 23.075 | 33.435 | 19.47 |
| Treatment (0.84 mg/kg) | 30.065 | 19.2 | 13.55 | 25.995 | 22.51 |
| Diane-35 | 12.185 | 21.525 | 22.995 | 11.275 | 16.57 |
